# Supplementary material for: Examining the association between family status and depression in the UK Biobank
Source: J Affect Disord. 2021 Jan 15;279:585–98. doi: 10.1016/j.jad.2020.10.017 (PMC7780845; doi:10.1016/j.jad.2020.10.017)
Supplement: Supplementary file 1 [file mmc1.docx]

**Supplementary material**

Table of contents

[Supplement 1 – Literature on covariates 3](#_Toc42530532)

[Supplement 2 – Depression phenotypes 10](#_Toc42530533)

[Supplement 3 – UK Biobank data fields 13](#_Toc42530534)

[Supplement 4 – Depression polygenic risk score 16](#_Toc42530535)

[Supplement 5 – ICD10 codes 17](#_Toc42530536)

[Supplement 6 – Medication codes 19](#_Toc42530537)

[Supplement 7 – Smith et al. mood disorders 21](#_Toc42530538)

[Supplement 8 – GWAS family status 22](#_Toc42530539)

[Supplement 9 – Mendelian randomisation 23](#_Toc42530540)

[Supplement 10 – SNP instruments 24](#_Toc42530541)

[Supplementary references 25](#_Toc42530542)

# Supplement 1 – Literature on covariates

**Table S1.** Literature documenting associations of covariates with depression and family status

| **Variable** | **Association with depression** | **Association with family status** |
| --- | --- | --- |
| **Age** | Kessler RC, Birnbaum HG, Shahly V, Bromet E, Hwang I, McLaughlin KA, et al. Age differences in the prevalence and co-morbidity of DSM-IV major depressive episodes: results from the WHO World Mental Health Survey Initiative. Depress Anxiety. 2010;27(4):351–64. | Bulloch AGM, Williams JVA, Lavorato DH, Patten SB. The depression and marital status relationship is modified by both age and gender. J Affect Disord. 2017 Dec 1;223:65–8. |
| **Sex** | Andrade L, Caraveo‐anduaga JJ, Berglund P, Bijl RV, Graaf RD, Vollebergh W, et al. The epidemiology of major depressive episodes: results from the International Consortium of Psychiatric Epidemiology (ICPE) surveys. Int J Methods Psychiatr Res. 2003;12(1):3–21.  Van de Velde S, Bracke P, Levecque K. Gender differences in depression in 23 European countries. Cross-national variation in the gender gap in depression. Soc Sci Med. 2010 Jul 1;71(2):305–13. | Simon RW. Revisiting the Relationships among Gender, Marital Status, and Mental Health. Am J Sociol. 2002;107(4):1065–96.  St John PD, Montgomery PR. Marital Status, Partner Satisfaction, and Depressive Symptoms in Older Men and Women. Can J Psychiatry. 2009 Jul 1;54(7):487–92.  Davila J, Karney BR, Hall TW, Bradbury TN. Depressive Symptoms and Marital Satisfaction: Within-Subject Associations and the Moderating Effects of Gender and Neuroticism. J Fam Psychol. 2003;17(4):557–70.  Jang S-N, Kawachi I, Chang J, Boo K, Shin H-G, Lee H, et al. Marital status, gender, and depression: Analysis of the baseline survey of the Korean Longitudinal Study of Ageing (KLoSA). Soc Sci Med. 2009 Dec 1;69(11):1608–15. |
| **Genetics** | Sullivan PF, Neale MC, Kendler KS. Genetic Epidemiology of Major Depression: Review and Meta-Analysis. Am J Psychiatry. 2000 Oct 1;157(10):1552–62.  Wray NR, Ripke S, Mattheisen M, Trzaskowski M, Byrne EM, Abdellaoui A, et al. Genome-wide association analyses identify 44 risk variants and refine the genetic architecture of major depression. Nat Genet. 2018 May;50(5):668.  Howard DM, Adams MJ, Clarke T-K, Hafferty JD, Gibson J, Shirali M, et al. Genome-wide meta-analysis of depression identifies 102 independent variants and highlights the importance of the prefrontal brain regions. Nat Neurosci. 2019 Mar;22(3):343. |  |
| **Death of spouse/partner** | Kamiya Y, Doyle M, Henretta JC, Timonen V. Depressive symptoms among older adults: The impact of early and later life circumstances and marital status. Aging Ment Health. 2013 Apr 1;17(3):349–57.  Jang S-N, Kawachi I, Chang J, Boo K, Shin H-G, Lee H, et al. Marital status, gender, and depression: Analysis of the baseline survey of the Korean Longitudinal Study of Ageing (KLoSA). Soc Sci Med. 2009 Dec 1;69(11):1608–15.  Van Grootheest DS, Beekman AT, Broese van Groenou MI, Deeg DJ. Sex differences in depression after widowhood. Do men suffer more? Soc Psychiatry Psychiatr Epidemiol. 1999 Jul;34(7):391–8. |  |
| **Marital separation/divorce** | Yan X-Y, Huang S-M, Huang C-Q, Wu W-H, Qin Y. Marital Status and Risk for Late Life Depression: A Meta-Analysis of the Published Literature. J Int Med Res. 2011 Aug 1;39(4):1142–54.  Andrade L, Caraveo‐anduaga JJ, Berglund P, Bijl RV, Graaf RD, Vollebergh W, et al. The epidemiology of major depressive episodes: results from the International Consortium of Psychiatric Epidemiology (ICPE) surveys. Int J Methods Psychiatr Res. 2003;12(1):3–21  Weissman MM, Bland RC, Canino GJ, Faravelli C, Greenwald S, Hwu H-G, et al. Cross-National Epidemiology of Major Depression and Bipolar Disorder. JAMA. 1996 Jul 24;276(4):293–9.  Kessler RC, Walters EE, Forthofer MS. The Social Consequences of Psychiatric Disorders, III: Probability of Marital Stability. Am J Psychiatry. 1998 Aug 1;155(8):1092–6.    Butterworth P, Rodgers B. Mental health problems and marital disruption: is it the combination of husbands and wives’ mental health problems that predicts later divorce? Soc Psychiatry Psychiatr Epidemiol. 2008 Sep 1;43(9):758–63. |  |
| **Migrant status** | Pernice R, Brook J. Relationship of Migrant Status (Refugee or Immigrant) to Mental Health. Int J Soc Psychiatry. 1994 Sep 1;40(3):177–88.  Riolo SA, Nguyen TA, Greden JF, King CA. Prevalence of Depression by Race/Ethnicity: Findings From the National Health and Nutrition Examination Survey III. Am J Public Health. 2005 Jun 1;95(6):998–1000. | Gazmararian JA, James SA, Lepkowski JM. Depression in black and white women: The role of marriage and socioeconomic status. Ann Epidemiol. 1995 Nov 1;5(6):455–63.  Almeida J, Subramanian SV, Kawachi I, Molnar BE. Is blood thicker than water? Social support, depression and the modifying role of ethnicity/nativity status. J Epidemiol Community Health. 2011 Jan;65(1):51–6. |
| **Highest educational/professional qualification** | Kessler RC, Foster CL, Saunders WB, Stang PE. Social consequences of psychiatric disorders, I: Educational attainment. Am J Psychiatry. 1995 Jul;152(7):1026–32 | Inaba A, Thoits PA, Ueno K, Gove WR, Evenson RJ, Sloan M. Depression in the United States and Japan: Gender, marital status, and SES patterns. Soc Sci Med. 2005 Dec 1;61(11):2280–92. |
| **Annual gross household income** | Levinson D, Lakoma MD, Petukhova M, Schoenbaum M, Zaslavsky AM, Angermeyer M, et al. Associations of serious mental illness with earnings: results from the WHO World Mental Health surveys. Br J Psychiatry. 2010 Aug;197(2):114–21.  McMillan KA, Enns MW, Asmundson GJG, Sareen J. The association between income and distress, mental disorders, and suicidal ideation and attempts: findings from the Collaborative Psychiatric Epidemiology Surveys. J Clin Psychiatry. 2010 Sep;71(9):1168–75.  Kawakami N, Abdulghani EA, Alonso J, Bromet EJ, Bruffaerts R, Caldas-de-Almeida JM, et al. Early-Life Mental Disorders and Adult Household Income in the World Mental Health Surveys. Biol Psychiatry. 2012 Aug 1;72(3):228–37.  Smith JP, Smith GC. Long-term economic costs of psychological problems during childhood. Soc Sci Med. 2010 Jul 1;71(1):110–5.  Muntaner C, Eaton WW, Miech R, O’Campo P. Socioeconomic Position and Major Mental Disorders. Epidemiol Rev. 2004 Jul 1;26(1):53–62. | LaPierre TA. The enduring effects of marital status on subsequent depressive symptoms among women: investigating the roles of psychological, social and financial resources. J Epidemiol Community Health. 2012 Nov 1;66(11):1056–62.  Jalovaara M. The joint effects of marriage partners’ socioeconomic positions on the risk of divorce. Demography. 2003 Feb 1;40(1):67–81. |
| **Employment status** | Dooley D, Fielding J, Levi L. Health and Unemployment. Annu Rev Public Health. 1996 Jan 1;17(1):449–65. | Loring Jones MSW A. The Relationship Between Unemployment and Divorce. J Divorce. 1989 Jan 18;12(1):99–112. |
| **Smoking status** | Kendler KS, Neale MC, MacLean CJ, Heath AC, Eaves LJ, Kessler RC. Smoking and Major Depression: A Causal Analysis. Arch Gen Psychiatry. 1993 Jan 1;50(1):36–43.  Chaiton MO, Cohen JE, O’Loughlin J, Rehm J. A systematic review of longitudinal studies on the association between depression and smoking in adolescents. BMC Public Health. 2009 Sep 22;9(1):356. | Broms U, Silventoinen K, Lahelma E, Koskenvuo M, Kaprio J. Smoking cessation by socioeconomic status and marital status: The contribution of smoking behavior and family background. Nicotine Tob Res. 2004 Jun 1;6(3):447–55.  Cho H-J, Khang Y-H, Jun H-J, Kawachi I. Marital status and smoking in Korea: The influence of gender and age. Soc Sci Med. 2008 Feb 1;66(3):609–19. |
| **Alcohol use** | Fergusson DM, Boden JM, Horwood LJ. Tests of Causal Links Between Alcohol Abuse or Dependence and Major Depression. Arch Gen Psychiatry. 2009 Mar 1;66(3):260–6.  90. Boden JM, Fergusson DM. Alcohol and depression. Addiction. 2011;106(5):906–14. | Prescott CA, Kendler KS. Associations between marital status and alcohol consumption in a longitudinal study of female twins. J Stud Alcohol. 2001 Jul 1;62(5):589–604.  Temple MT, Fillmore KM, Hartka E, Johnstone B, Leino EV, Motoyoshi M. A meta-analysis of change in marital and employment status as predictors of alcohol consumption on a typical occasion*. Br J Addict. 1991;86(10):1269–81.  Power C, Rodgers B, Hope S. Heavy alcohol consumption and marital status: disentangling the relationship in a national study of young adults. Addiction. 1999;94(10):1477–87. |
| **Neuroticism** | Okbay A, Baselmans BML, De Neve J-E, Turley P, Nivard MG, Fontana MA, et al. Genetic variants associated with subjective well-being, depressive symptoms, and neuroticism identified through genome-wide analyses. Nat Genet. 2016 Jun;48(6):624–33.  Ormel J, Jeronimus BF, Kotov R, Riese H, Bos EH, Hankin B, et al. Neuroticism and Common Mental Disorders: Meaning and Utility of a Complex Relationship. Clin Psychol Rev. 2013 Jul;33(5):686–97. | Cramer D. Personality and marital dissolution. Personal Individ Differ. 1993 Apr 1;14(4):605–7.  Kelly EL, Conley JJ. Personality and compatibility: a prospective analysis of marital stability and marital satisfaction. J Pers Soc Psychol. 1987 Jan;52(1):27–40. |
| **Long-standing illness, disability or infirmity** | Turner RJ, Noh S. Physical Disability and Depression: A Longitudinal Analysis. J Health Soc Behav. 1988;29(1):23–37. | Caputo J, Simon RW. Physical Limitation and Emotional Well-Being: Gender and Marital Status Variations. J Health Soc Behav. 2013 Jun 1;54(2):241–57. |
| **Adverse childhood experiences** | Maniglio R. Child sexual abuse in the etiology of depression: A systematic review of reviews. Depress Anxiety. 2010;27(7):631–42.  Nanni V, Uher R, Danese A. Childhood maltreatment predicts unfavorable course of illness and treatment outcome in depression: a meta-analysis. Am J Psychiatry. 2012 Feb;169(2):141–51.  Nguyen TP, Karney BR, Bradbury TN. Childhood abuse and later marital outcomes: Do partner characteristics moderate the association? J Fam Psychol JFP J Div Fam Psychol Am Psychol Assoc Div 43. 2017 Feb;31(1):82–92. | Larson JH, LaMont C. The Relationship of Childhood Sexual Abuse to the Marital Attitudes and Readiness for Marriage of Single Young Adult Women. J Fam Issues. 2005 May 1;26(4):415–30. |
| **Traumatic life events** | Mazure CM. Life Stressors as Risk Factors in Depression. Clin Psychol Sci Pract. 1998 Sep 1;5(3):291–313.  Clarke T-K, Zeng Y, Navrady L, Xia C, Haley C, Campbell A, et al. Genetic and environmental determinants of stressful life events and their overlap with depression and neuroticism. Wellcome Open Res. 2019 Jan 14;3:11.  Cairns KE, Yap MBH, Pilkington PD, Jorm AF. Risk and protective factors for depression that adolescents can modify: A systematic review and meta-analysis of longitudinal studies. J Affect Disord. 2014 Dec 1;169:61–75. | Finkelhor D, Hotaling GT, Lewis IA, Smith C. Sexual Abuse and Its Relationship to Later Sexual Satisfaction, Marital Status, Religion, and Attitudes. J Interpers Violence. 1989 Dec 1;4(4):379–99. |
| **Participation in social/leisure activities** | Dunn A, Trivedi M, O’neal H. Physical activity dose-response effects on outcomes of depression and anxiety. Med Sci Sports Exerc [Internet]. 2001 Jun 1 [cited 2019 May 13];33(6).  Kvam S, Kleppe CL, Nordhus IH, Hovland A. Exercise as a treatment for depression: A meta-analysis. J Affect Disord. 2016 Sep 15;202:67–86. | Janke MC, Nimrod G, Kleiber DA. Leisure Activity and Depressive Symptoms of Widowed and Married Women in Later Life. J Leis Res. 2008 Jun 1;40(2):250–66.  Wang F, DesMeules M, Luo W, Dai S, Lagace C, Morrison H. Leisure-time physical activity and marital status in relation to depression between men and women: A prospective study. Health Psychol. 2011;30(2):204–11. |
| **Loneliness** | Matthews T, Danese A, Wertz J, Odgers CL, Ambler A, Moffitt TE, et al. Social isolation, loneliness and depression in young adulthood: a behavioural genetic analysis. Soc Psychiatry Psychiatr Epidemiol. 2016 Mar 1;51(3):339–48.  Dill JC, Anderson CA. Loneliness, shyness, and depression: The etiology and interrelationships of everyday problems in living. In: The interactional nature of depression: Advances in interpersonal approaches. Washington, DC, US: American Psychological Association; 1999. p. 93–125.  Heinrich LM, Gullone E. The clinical significance of loneliness: A literature review. Clin Psychol Rev. 2006 Oct 1;26(6):695–718. | Stack S. Marriage, Family and Loneliness: A Cross-National Study. Sociol Perspect. 1998 Jun 1;41(2):415–32. |
| **Ever had same-sex intercourse** | Bailey JM. Homosexuality and Mental Illness. Arch Gen Psychiatry. 1999 Oct 1;56(10):883–4.  Cochran SD, Mays VM. Lifetime prevalence of suicide symptoms and affective disorders among men reporting same-sex sexual partners: results from NHANES III. Am J Public Health. 2000 Apr;90(4):573–8.  Gilman SE, Cochran SD, Mays VM, Hughes M, Ostrow D, Kessler RC. Risk of psychiatric disorders among individuals reporting same-sex sexual partners in the National Comorbidity Survey. Am J Public Health. 2001 Jun;91(6):933–9.  Zietsch BP, Verweij KJH, Heath AC, Madden P a. F, Martin NG, Nelson EC, et al. Do shared etiological factors contribute to the relationship between sexual orientation and depression? Psychol Med. 2012 Mar;42(3):521–32.  Marshal MP, Dietz LJ, Friedman MS, Stall R, Smith HA, McGinley J, et al. Suicidality and Depression Disparities Between Sexual Minority and Heterosexual Youth: A Meta-Analytic Review. J Adolesc Health. 2011 Aug 1;49(2):115–23. |  |
| **Number of lifetime sexual partners** | Khan MR, Kaufman JS, Pence BW, Gaynes BN, Adimora AA, Weir SS, et al. Depression, Sexually Transmitted Infection, and Sexual Risk Behavior Among Young Adults in the United States. Arch Pediatr Adolesc Med. 2009 Jul 6;163(7):644–52.  Lehrer JA, Shrier LA, Gortmaker S, Buka S. Depressive Symptoms as a Longitudinal Predictor of Sexual Risk Behaviors Among US Middle and High School Students. Pediatrics. 2006 Jul 1;118(1):189–200. | Rector R, A Johnson K, D Lauren P, Noyes R, Martin S. The Harmful Effects of Early Sexual Activity and Multiple Sexual Partners among Women: A Book of Charts. 2002 Jan 1;  Teachman J. Premarital Sex, Premarital Cohabitation, and the Risk of Subsequent Marital Dissolution Among Women. J Marriage Fam. 2003;65(2):444–55. |
| **Body mass index** | De Wit LM, van Straten A, van Herten M, Penninx BW, Cuijpers P. Depression and body mass index, a u-shaped association. BMC Public Health. 2009 Jan 13;9(1):14.  Ho RCM, Niti M, Kua EH, Ng T-P. Body mass index, waist circumference, waist–hip ratio and depressive symptoms in Chinese elderly: a population-based study. Int J Geriatr Psychiatry. 2008;23(4):401–8. | Sobal J, Rauschenbach BS, Frongillo EA. Marital status, fatness and obesity. Soc Sci Med. 1992 Oct 1;35(7):915–23  Jeffery RW, Rick AM. Cross-Sectional and Longitudinal Associations between Body Mass Index and Marriage-Related Factors. Obes Res. 2002;10(8):809–15. |

# Supplement 2 – Depression phenotypes

**Table S2.** Depression phenotypes.

| **Variable** | **Definition + UK Biobank data fields** |
| --- | --- |
| **Lifetime depression** (case):  At least one core symptom of major depressive disorder, most or all of the day on most or all days for a two week period, with at least five non-core depressive symptoms that represent a change from usual occurring over the same time-scale, with some or a lot of impairment.  No self-reported psychosis or mania. | ("Ever had prolonged feelings of sadness or depression" (20446) = Yes OR "Ever had prolonged loss of interest in normal activities" (20441) = Yes)  AND  "Fraction of day affected during worst episode of depression" (20436) = Most of day or All day long  AND  "Frequency of depressed days during worst episode of depression" (20439) = "Almost every day" or "Every day"  AND "Impact on normal roles during worst period of depression" (20440) = "Somewhat" or "A lot"  AND  Total number of symptoms endorsed (core and others) ≥5:  "Ever had prolonged feelings of sadness or depression" (core) (20446), "Ever had prolonged loss of interest in normal activities" (core) (20441), "Feelings of tiredness during worst episode of depression" (20449), "Weight change during worst episode of depression" (20536), "Did your sleep change?" (20532), "Difficulty concentrating during worst depression (20435), "Feelings of worthlessness during worst period of depression" (20450), "Thoughts of death during worst depression" (20437)  AND  No self-reported psychosis or mania for "Mental health problems ever diagnosed by a professional" (20544). |
| **Lifetime depression** (control):  No self-reported diagnosis of depression or screening positive on CIDI-SF or PHQ-9.  No self-reported psychosis or mania or any other mental disorder, not currently taking antidepressant medications, no mood disorder hospital episode record and not meeting Smith et al. criteria for mood disorder. | No self-reported diagnosis of depression (20544 and 20002)  AND  No core symptom as described above and not meeting above criteria for duration and level of impairment  AND  PHQ-9 sum score <5 (see details below)  AND  No self-reported psychosis, mania or any other mental disorder for "Mental health problems ever diagnosed by a professional" (20544), an no addiction ("Ever addicted to any substance or behaviour" (20401) = "No", "Maximum frequency of taking cannabis" (20454) less than "every day" for those indicating "Ever taken cannabis" (20453) = "Yes")  AND  Not currently taking antidepressant medication (see Supplement 5)  AND  No mood disorder hospital episode record (see Supplement 6)  AND  Not meeting Smith et al. criteria for mood disorder (20126; see Supplement 7). |
| **Lifetime severe depression** (case):  Lifetime depression (case) and endorsing all CIDI-SF symptoms, with a lot of impact on normal roles during worst periods of depression. | Meeting criteria for lifetime depression (case) as described above  AND  CIDI-SF score = 8 (i.e. all symptoms endorsed)  AND  "Impact on normal roles during worst period of depression" (20440) = "A lot". |
| **Lifetime severe depression** (control):  Lifetime depression (control) and not including individuals who met criteria for lifetime depression (case) but not for lifetime severe depression (case). | Meeting criteria for lifetime depression (control) as described above  AND  Excluding individuals meeting criteria for lifetime depression (case) but not lifetime severe depression (case). |
| **Current depression** (case):  Lifetime depression (case) and endorsing five or more symptoms (including at least one core symptom) during the past two weeks at least “more than half the days” for first eight items and at least “several days” for last item. | Meeting criteria for lifetime depression (case) as described above  AND  Total PHQ-9 symptoms endorsed occurring "more than half the days" or "nearly every day" (for "Recent thoughts of suicide or self-harm" (20513) also including "several days") ≥ 5, including ≥1 core symptom occurring "more than half the days" or "nearly every day":  "Recent lack of interest or pleasure in doing things" (core) (20514), “Recent feelings of depression” (core) (20510), "Trouble falling or staying asleep, or sleeping too much" (20517), "Recent feelings of tiredness or low energy" (20519), "Recent poor appetite or overeating" (20511), "Recent feelings of inadequacy" (20507), "Recent trouble concentrating on things" (20508), "Recent changes in speed/amount of moving or speaking" (20518), "Recent thoughts of suicide or self-harm" (20513). |
| **Current depression** (control):  Lifetime depression (control) and PHQ-9 sum score of less than five (cut-off for "mild depression"). | Meeting criteria for lifetime depression (control) as described above  AND  PHQ-9 sum score <5. |
| **Current severe depression** (case):  Current depression (case) and PHQ-9 sum score of more than 14. | Meeting criteria for current depression (case) as described above  AND  PHQ-9 sum score >14. |
| **Current severe depression** (control):  Current depression (control) and not including individuals who met criteria for current depression (case) but not for current severe depression (case). | Meeting criteria for current depression (control) as described above  AND  Excluding individuals meeting criteria for current depression (case) but not current severe depression (case). |
| **PHQ-9 sum score:**  Score all PHQ-9 items 0-3 and sum. | Sum ("Recent lack of interest or pleasure in doing things" (core) (20514), “Recent feelings of depression” (core) (20510), "Trouble falling or staying asleep, or sleeping too much" (20517), "Recent feelings of tiredness or low energy" (20519), "Recent poor appetite or overeating" (20511), "Recent feelings of inadequacy" (20507), "Recent trouble concentrating on things" (20508), "Recent changes in speed/amount of moving or speaking" (20518), "Recent thoughts of suicide or self-harm" (20513)).  (Subtract 9 if items scored 1-4).  If value missing, count as “0” when scoring 0-3. |
| *Note:* Depression phenotypes adapted from Davis et al (2020). | |

# Supplement 3 – UK Biobank data fields

**Table S3.** Phenotypes calculated based on UK Biobank data-fields.

| **Variable** | **UK Biobank fields** |
| --- | --- |
| **Age at completing the MHQ** | "Age when attended assessment centre" (21003) + (("Date of completing mental health questionnaire" (20400) – "Date of attending assessment centre"(53))/365.25 |
| **Age group** | Above divided into five categories:  "45-54",  "55-64",  "65-74",  "75-80" |
| **Sex** | "Sex" (31):  "Female",  "Male" |
| **Death of a spouse/partner** (in the two years prior to assessment) | "Death of spouse or partner" for "Illness, bereavement, stress in the past two years" (6145):  "Yes",  "No" |
| **Marital separation/divorce** (in the two years prior to assessment) | "Marital separation/divorce" for "Illness, bereavement, stress in the past two years" (6145):  "Yes",  "No" |
| **Migrant status** | "Country of birth (UK/elsewhere)" (1647):  "Native" (i.e. born in the UK),  "Migrant" (i.e. born in Ireland or elsewhere) |
| **Highest educational or professional qualification** | “Qualifications” (6138) divided into four categories:  "Degree",  "A levels/NVQ/HND/HNC/other professional qualifications",  "O levels/GCSEs/CSEs",  "None of the above" |
| **Annual gross household income** | "Average total household income before tax" (738):  "Less than £18,000",  "£18,000 to £30,999",  "£31,000 to £51,999",  "£52,000 to £100,000",  "Greater than £100,000" |
| **Employment status** | "Current employment status" (6142) divided into three categories:  "Employed" (i.e. "in paid employment or self-employed"),  "Unemployed",  "Inactive" (i.e. "unable to work", "looking after home or family", "doing unpaid or voluntary work", "full or part-time student" or "retired") |
| **Townsend deprivation index** | "Townsend deprivation index at recruitment" (189) |
| **Smoking status** | "Smoking status" (20116):  "Never",  "Former",  "Current" |
| **Alcohol use** | "Amount of alcohol drunk on a typical drinking day" (20403), "Ever had known person concerned about, or recommend reduction of, alcohol consumption" (20405), "Frequency of failure to fulfil normal expectations due to drinking alcohol in last year" (20407), "Frequency of memory loss due to drinking alcohol in last year" (20408), "Frequency of feeling guilt or remorse after drinking alcohol in last year" (20409), "Ever been injured or injured someone else through drinking alcohol" (20411), "Frequency of needing morning drink of alcohol after heavy drinking session in last year" (20412), "Frequency of inability to cease drinking in last year" (20413), "Frequency of drinking alcohol" (20414), "Frequency of consuming six or more units of alcohol" (20416)  Alcohol Use Disorders Identification Test (AUDIT) score derived from variables listed above (43):  "Normal" (i.e. 0-7),  "Hazardous" (i.e. 8-15),  "Harmful" (i.e. 16-19),  "Dependent" (i.e. 20+) |
| **Neuroticism** | "Neuroticism score" (20127) |
| **Long-standing illness, disability or infirmity** | "Long-standing illness, disability or infirmity" (2188):  "Yes",  "No" |
| **Adverse childhood experiences** | "Felt hated by family member" (20487), "Physically abused by family" (20488), "Felt loved" (20489), "Sexually molested" (20490), "Had someone to take to doctor when needed" (20491) combined into two categories:  "Yes" (i.e. if any of the following: 20487 = "sometimes true", "often" or "very often true"; 20488 = "rarely true", "sometimes true", "often" or "very often true"; 20489 = "never true", "rarely true" or "sometimes true"; 20490 = "rarely true", "sometimes true", "often" or "very often true"; 20491 = "never true", "rarely true" or "sometimes true"),  "No" (i.e. not fulfilling criteria above) |
| **Traumatic life events** | "Been in serious accident believed to be life-threatening" (20526), "Been involved in combat or exposed to war-zone" (20527), "Diagnosed with life-threatening illness" (20528), "Victim of physically violent crime" (20529), "Witnessed sudden violent death" (20530), "Victim of sexual assault" (20531) combined into two categories:  "Yes" (i.e. "yes, within the last 12 months" or "yes, but not in the last 12 months" for any of the above),  "No" (i.e. "never" for all of the above) |
| **Participation in leisure/social activities** | "Leisure/social activities" (6160) divided into two categories:  "Yes" (i.e. involved in "sports club or gym", "pub or social club", "religious group", "adult education class" or "other group activity")  "No" (i.e. "none of the above") |
| **Loneliness*** | "Do you often feel lonely?" (2020), "How often are you able to confide in someone close to you?" (2110) combined into two categories:  "Yes" (i.e. 2020 = "yes" and 2110 = "never or almost never" or "once every few months"),  "No" (not fulfilling criteria above) |
| **Ever had same-sex intercourse** | "Ever had same-sex intercourse" (2159):  "Yes",  "No" |
| **Lifetime number of sexual partners** | "Lifetime number of sexual partners" (2149) |
| **Body mass index** | "Body mass index (BMI)" (21001) |

***Loneliness index:**

1. If the response to one question was missing, “don’t know” or “prefer not to answer” and the response to the second question was scored 0, individuals were classified as not lonely.
2. If the response to one question was missing, “don’t know” or “prefer not to answer” and the response to the second question was scored 1, individuals were classified as missing data on loneliness.
3. If responses to both questions were missing, “don’t know” or “prefer not to answer”, individuals were classified as missing data on loneliness.

# Supplement 4 – Depression polygenic risk score

**Depression PRS based on summary statistics from** Wray et al. (2018).

Individuals were excluded where recommended by the UK Biobank core analysis team for unusual levels of missingness or heterozygosity, or if they had withdrawn consent for analysis. Using the genotyped SNPs, individuals with call rate <98%, who were related to another individual in the dataset (KING *r* < 0.044, equivalent to removing third-degree relatives and closer (Manichaikul et al. 2010) or whose self-reported and genotypic sex information was discordant (X-chromosome homozygosity (*F*_X_) < 0.9 for phenotypic males, *F*_X_ > 0.5 for phenotypic females) were also excluded. Removal of relatives was performed using a “greedy” algorithm, which minimises exclusions (for example, by excluding the child in a mother–father–child trio). All analyses were limited to individuals of European ancestry, as defined by 4-means clustering on the first two genetic principal components provided by the UK Biobank (Warren et al. 2017). This ancestry group included 95% of the respondents to the mental health questionnaire—as such, the non-European ancestry groups were considered too small to analyse informatively. Principal components analysis was also performed on the European-only subset of the data using the software flashpca2 (Abraham et al. 2017).

Depression PRS were constructed using PRSice v2 (Choi & O'Reilly, 2019) in unrelated individuals of European ancestry using the genotype data and quality control procedures described above. Summary statistics from Wray et al. (2018) with 23andMe and UK Biobank samples removed (N_cases_ = 45,591, N_controls_ = 97,674) were used as the base dataset. To account for linkage disequilibrium, clumping was performed so that single nucleotide polymorphisms (SNPs) had an *R^2^* < 0.1 and a 250kb window from other SNPs. Depression PRS were then calculated across 11 SNP significance thresholds (*p* < 5 × 10−8, *p* < 1 × 10−5, *p* < 0.001, *p* < 0.01, *p* < 0.05, *p* < 0.1, *p* < 0.2, *p* < 0.3, *p* < 0.4, *p* < 0.5, *p* < 1). Before fitting the full model, we ran separate regressions with depression PRS generated at different SNP significance thresholds, with principal components and batches as covariates and lifetime depression as the dependent variable. The PRS at a p-value threshold of 0.3 yielded the highest Nagelkerke R^2^ (0.0167) and is the one we included in our analyses.

# Supplement 5 – ICD10 codes

ICD10 codes for primary or secondary mood disorder diagnoses (Hospital Episode Statistics)

**Manic episode**

- F30.0 Hypomania
- F30.1 Mania without psychotic symptoms
- F30.2 Mania with psychotic symptoms
- F30.8 Other manic episodes
- F30.9 Manic episode, unspecified

**Bipolar affective disorder**

- F31.0 Bipolar affective disorder, current episode hypomanic
- F31.1 Bipolar affective disorder, current episode manic without psychotic symptoms
- F31.2 Bipolar affective disorder, current episode manic with psychotic symptoms
- F31.3 Bipolar affective disorder, current episode mild or moderate depression
- F31.4 Bipolar affective disorder, current episode severe depression without psychotic symptoms
- F31.5 Bipolar affective disorder, current episode severe depression with psychotic symptoms
- F31.6 Bipolar affective disorder, current episode mixed
- F31.7 Bipolar affective disorder, currently in remission
- F31.8 Other bipolar affective disorders
- F31.9 Bipolar affective disorder, unspecified

**Depressive episode**

- F32.0 Mild depressive episode
- F32.1 Moderate depressive episode
- F32.2 Severe depressive episode without psychotic symptoms
- F32.3 Severe depressive episode with psychotic symptoms
- F32.8 Other depressive episodes
- F32.9 Depressive episode, unspecified

**Recurrent depressive disorder**

- F33.0 Recurrent depressive disorder, current episode mild
- F33.1 Recurrent depressive disorder, current episode moderate
- F33.2 Recurrent depressive disorder, current episode severe without psychotic symptoms
- F33.3 Recurrent depressive disorder, current episode severe with psychotic symptoms
- F33.4 Recurrent depressive disorder, currently in remission
- F33.8 Other recurrent depressive disorders
- F33.9 Recurrent depressive disorder, unspecified

**Persistent mood [affective] disorders**

- F34.0 Cyclothymia
- F34.1 Dysthymia
- F34.8 Other persistent mood [affective] disorders
- F34.9 Persistent mood [affective] disorder, unspecified

**Other mood [affective] disorders**

- F38.0 Other single mood [affective] disorders
- F38.1 Other recurrent mood [affective] disorders
- F38.8 Other specified mood [affective] disorders
- F39 Unspecified mood [affective] disorder

# Supplement 6 – Medication codes

Self-reported medication codes

- 1140879616 Amitriptyline
- 1140921600 Citalopram
- 1140879540 Fluoxetine
- 1140867878 Sertraline
- 1140916282 Venlafaxine
- 1140909806 Dosulepin
- 1140867888 Paroxetine
- 1141152732 Mirtazapine
- 1141180212 Escitalopram
- 1140879634 Trazodone
- 1140867876 Prozac 20mg Capsule
- 1140882236 Seroxat 20mg Tablet
- 1141190158 Cipralex 5mg Tablet
- 1141200564 Duloxetine
- 1140867726 Lofepramine
- 1140879620 Clomipramine
- 1140867818 Nortriptyline
- 1140879630 Imipramine
- 1140879628 Dothiepin
- 1141151946 Cipramil 10mg Tablet
- 1140867948 Amitriptyline Hydrochloride+Perphenazine 10mg/2mg Tablet
- 1140867624 Prothiaden 25mg Capsule
- 1140867756 Trimipramine
- 1140867884 Lustral 50mg Tablet
- 1141151978 Reboxetine
- 1141152736 Zispin 30mg Tablet
- 1141201834 Cymbalta 30mg Gastro-Resistant Capsule
- 1140867690 Anafranil 10mg Capsule
- 1140867640 Doxepin
- 1140867920 Moclobemide
- 1140867850 Phenelzine
- 1140879544 Fluvoxamine
- 1141200570 Yentreve 20mg Gastro-Resistant Capsule
- 1140867934 Triptafen Tablet
- 1140867758 Surmontil 10mg Tablet
- 1140867914 Tranylcypromine
- 1140867820 Allegron 10mg Tablet
- 1141151982 Edronax 4mg Tablet
- 1140882244 Molipaxin 50mg Capsule
- 1140879556 Mianserin
- 1140867852 Nardil 15mg Tablet
- 1140867860 Faverin 50mg Tablet
- 1140917460 Nefazodone
- 1140867938 Amitriptyline+Chlordiazepoxide 12.5mg/5mg Capsule
- 1140867856 Isocarboxazid
- 1140867922 Manerix 150mg Tablet
- 1140910820 Maoi – Tranylcypromine
- 1140882312 Sinequan 10mg Capsule
- 1140867944 Tranylcypromine+Trifluoperazine 10mg/1mg Tablet
- 1140867784 Ludiomil 10mg Tablet
- 1140867812 Norval 10mg Tablet
- 1140867668 Tryptizol 10mg Tablet
- 1140867940 Fluphenazine Hydrochloride+Nortriptyline 1.5mg/30mg Tablet

# Supplement 7 – Smith et al. mood disorders

**Mood disorders adapted from** Smith et al. (2013).

**1. Probable bipolar disorder (type I):**

4642 ever manic/hyper 2 days OR 4653 ever irritable/argumentative for 2 days;

plus at least 3 from 6156.01 (more active), 6156.02 (more talkative), 6156.03 (needed less sleep), and 6156.04 (more creative/more ideas);

plus 5663 duration of a week or more;

plus 5674 needed treatment or caused problems at work.

**2. Probable bipolar disorder (type II):**

4642 ever manic/hyper 2 days OR 4653 ever irritable/argumentative for 2 days;

plus at least 3 from 6156.01 (more active), 6156.02 (more talkative), 6156.03 (needed less sleep), and 6156.04 (more creative/more ideas);

plus 5663 duration of a week or more.

**3. Single probable episode of major depression:**

4598 ever depressed/down for a whole week; plus 4609 at least two weeks duration; plus

4620 only one episode, plus 2090 ever seen a GP or 2100 a psychiatrist for nerves, anxiety, depression

OR

4631 ever anhedonic (unenthusiasm/uninterest) for a whole week; plus 5375 at least two weeks; plus

5386 only one episode; plus 2090 ever seen a GP or 2100 a psychiatrist for nerves, anxiety, depression.

**4. Probable recurrent major depression (moderate):**

4598 ever depressed/down for a whole week; plus 4609 at least two weeks duration; plus 4620 at least two episodes; plus 2090 ever seen a GP (but not a psychiatrist) for nerves, anxiety, depression

OR

4631 ever anhedonic (unenthusiasm/uninterest) for a whole week; plus 5375 at least two weeks; plus

5386 at least two episodes; plus 2090 ever seen a GP (but not a psychiatrist) for nerves, anxiety, depression.

**5. Probable recurrent major depression (severe):**

4598 ever depressed/down for a whole week; plus 4609 at least two weeks duration; plus 4620 at least two episodes; plus 2100 ever seen a psychiatrist for nerves, anxiety, depression

OR

4631 ever anhedonic (unenthusiasm/uninterest) for a whole week; plus 5375 at least two weeks; plus

5386 at least two episodes; plus 2100 ever seen a psychiatrist for nerves, anxiety, depression.

# Supplement 8 – GWAS family status

**Genome-wide association studies family status**

Two genome-wide association studies (GWAS) were carried out using UK Biobank data. The outcome variable in the first GWAS was number of children defined as an ordinal variable with the same levels as in the phenotypic analyses. The sample size for this analysis was *n* = 385 053. In the second GWAS the outcome variable was cohabitation with a spouse or partner as described in the phenotypic analyses. The sample size for this analysis was *n* = 311 942. Covariates including six ancestry-informative principal components, batch number and assessment centre were regressed out of each phenotype using logistic regression for cohabitation status and Poisson regression for number of children. The resulting residuals were used as the dependent variables for performing GWAS using BGENIE v1.2 software (Bycroft et al., 2018). For lifetime depression, we used the latest MDD Psychiatric Genomics Consortium (PGC) GWAS, excluding data from UK Biobank or 23andMe, resulting in a sample of 45 396 individuals with clinically diagnosed MDD and 97 250 controls (Wray et al, 2018).

# Supplement 9 – Mendelian randomisation

| **Table S5.** Mendelian randomisation; alternative methods. | | | | | | | |
| --- | --- | --- | --- | --- | --- | --- | --- |
| Method | *β* | | SE | 95% confidence interval | | p-value | Number of SNPs |
| **Number of children on lifetime depression** | | | | | | | |
| IVW | 0.6843 | 0.3326 | | 0.0325 | 1.3361 | 0.0396 | 21 |
| MR-RAPS | 0.7182 | 0.3426 | | 0.0467 | 0.2509 | 0.0360 | 21 |
| MR-Egger | -0.3170 | 1.5677 | | -3.3896 | 2.7556 | 0.8419 | 21 |
| Weighted Median | 0.4605 | 0.3764 | | -0.2773 | 1.1982 | 0.2212 | 21 |
| **Cohabitation status on lifetime depression** | | | | | | | |
| IVW | -0.2655 | 0.4117 | | -1.0725 | 0.5415 | 0.5190 | 18 |
| MR-RAPS | -0.2691 | 0.4993 | | -1.2477 | 2.9447 | 0.5899 | 18 |
| MR-Egger | -1.5676 | 4.0913 | | -9.5863 | 6.4512 | 0.7016 | 18 |
| Weighted Median | -0.4166 | 0.5741 | | -1.5418 | 0.7087 | 0.4681 | 18 |
| **Lifetime depression on number of children** | | | | | | | |
| IVW | 0.0042 | 0.0088 | | -0.0130 | 0.0213 | 0.6353 | 12 |
| MR-RAPS | 0.0044 | 0.0079 | | -0.0112 | 0.0298 | 0.5818 | 12 |
| MR-Egger | -0.0043 | 0.0215 | | -0.0463 | 0.0377 | 0.8411 | 12 |
| Weighted Median | -0.0001 | 0.0090 | | -0.0177 | 0.0174 | 0.9900 | 12 |
| **Lifetime depression on cohabitation status** | | | | | | | |
| IVW | 0.0067 | 0.0069 | | -0.0068 | 0.0202 | 0.3315 | 12 |
| MR-RAPS | 0.0074 | 0.0075 | | -0.0072 | 0.0216 | 0.3196 | 12 |
| MR-Egger | -0.0064 | 0.0163 | | -0.0384 | 0.0256 | 0.6949 | 12 |
| Weighted Median | 0.0042 | 0.0097 | | -0.0148 | 0.0232 | 0.6637 | 12 |

# Supplement 10 – SNP instruments

| **Table S6.** SNPs used for MR analyses, by association with each exposure. | | |
| --- | --- | --- |
| **Number of children** | **Cohabitation status** | **Lifetime depression** |
| rs56031423 | rs12600080 | rs1950829 |
| rs1913475 | rs4407923 | rs76025409 |
| rs11987838 | rs3740373 | rs6832890 |
| rs1899048 | rs79261179 | rs17499892 |
| rs7793877 | rs58130365 | rs1936365 |
| rs13213141 | rs113237425 | rs1460943 |
| rs12888512 | rs28459132 | rs4811079 |
| rs1880206 | rs73121943 | rs12129573 |
| rs6800021 | rs73600401 | rs2451828 |
| rs35091253 | rs17574906 | rs10825942 |
| rs66766977 | rs73347054 | rs1025145 |
| rs9421249 | rs77762196 | rs78676209 |
| rs12203592 | rs11213279 |  |
| rs12932628 | rs61762852 |  |
| rs824394 | rs1475903 |  |
| rs227361 | rs117372337 |  |
| rs16930736 | rs2630419 |  |
| rs12487989 | rs56982541 |  |
| rs9815134 |  |  |
| rs75555358 |  |  |
| rs2802282 |  |  |

# Supplementary references

Abraham, G., Qiu, Y., Inouye, M. (2017). FlashPCA2: principal component analysis of Biobank-scale genotype datasets. Bioinformatics, 33, 2776–2778.

Bycroft, C., Freeman, C., Petkova, D., Band, G., Elliott, L. T., Sharp, K., ... & Cortes, A. (2018). The UK Biobank resource with deep phenotyping and genomic data. Nature, 562(7726), 203-209.

Coleman, J. R., Peyrot, W. J., Purves, K. L., Davis, K. A., Rayner, C., Choi, S. W., ... Breen, G. (2020). Genome-wide gene-environment analyses of major depressive disorder and reported lifetime traumatic experiences in UK Biobank. Molecular psychiatry, 1-17.

Choi, S. W., & O'Reilly, P. F. (2019). PRSice-2: Polygenic Risk Score software for biobank-scale data. Gigascience, 8(7).

Davis, K. A., Coleman, J. R., Adams, M., Allen, N., Breen, G., Cullen, B., ... Hotopf, M. (2020). Mental health in UK Biobank–development, implementation and results from an online questionnaire completed by 157 366 participants: a reanalysis. BJPsych open, 6(2).

Manichaikul, A., Mychaleckyj, J.C., Rich, S.S., Daly, K., Sale, M., Chen, W.M. (2010) Robust relationship inference in genome-wide association studies. Bioinformatics, 26(22), 2867-73.

Warren, H.R., Evangelou, E., Cabrera, C.P., Gao, H., Ren, M., Mifsud, B. ... UK Biobank CardioMetabolic Consortium BP working group. (2017). Genome-wide association analysis identifies novel blood pressure loci and offers biological insights into cardiovascular risk. Nature genetics, 49(3), 403– 415.

Wray NR, Ripke S, Mattheisen M, Trzaskowski M, Byrne EM, Abdellaoui A, et al. Genome-wide association analyses identify 44 risk variants and refine the genetic architecture of major depression. Nat Genet. 2018 May;50(5):668.
